# Salzmann nodular degeneration in posterior keratoconus: a case report

**DOI:** 10.1186/s12886-022-02268-3

**Published:** 2022-03-12

**Authors:** Peng Song, Xiaofei Yu, Chenjiu Pang

**Affiliations:** Department of Ophthalmology, Henan Provincial People’s Hospital, Henan Eye Institute, People’s Hospital of Zhengzhou University, Weiwu Road 7, Zhengzhou, 450003 China

**Keywords:** Salzmann nodular degeneration, Posterior keratoconus, Phototherapeutic keratectomy

## Abstract

**Background:**

To report an unusual case of salzmann nodular degeneration (SND) in posterior keratoconus (PKC) after a corneal penetrating injury.

**Case presentation:**

A 56-year-old woman presented with a history of recurrent light sensitivity, foreign body sensation, and tears after a corneal penetrating injury 20 years ago. The patient was diagnosed with SND accompanying with PKC by slit-lamp microscope, anterior segment optical coherence tomography (OCT), and corneal tomography. A combined therapy of medication (0.1% sodium hyaluronate eye drops, recombinant bovine basic fibroblast growth factor eye drops, and 0.1% fluorometholone eye drops) and bandage contact lens could not relieve the latest episode. A phototherapeutic keratectomy (PTK) treatment (laser ablation depth: 15 μm; treatment zone: 7.5 mm) was performed to remove nodules and smooth the surface. The best spectacle-corrected visual acuity improved from 20/63 preoperatively to 20/40 postoperatively. No SND relapse and corneal ectasia were recorded at follow-up 12 months later.

**Conclusions:**

This is the first known, reported case of SND accompanying with PKC after corneal trauma. The PTK is a safe and effective option for SND with PKC.

## Background

Salzmann nodular degeneration (SND) is a rare, noninflammatory, and progressive corneal degenerative condition that is commonly characterized by elevated, whitish-grey nodules located beneath thinned corneal epithelium [1, 2]. The exact etiology is still unknown, but evidence points to chronic ocular surface inflammation secondary to trachoma, interstitial keratitis, dry eye, and ocular trauma playing an important role in SND developing [1]. Additionally, familial studies and case series have demonstrated both genetic and environmental factors as probable causes of SND [2]. Previous studies suggested that the histologic features of SND include attenuated epithelium, disruption of the Bowman layer, fibroblast overgrowth, and deposition of the extracellular matrix [3–5]. Posterior keratoconus (PKC) is a rare corneal developmental anomaly characterized by an entire or localized abnormality of the posterior corneal surface [6]. Also, cases acquired after trauma have been reported [7].

In this study, we describe a case of SND accompanied with PKC after corneal penetrating injury. A phototherapeutic keratectomy (PTK) was applied to remove the nodules and prevent SND recurrence. To our knowledge, this is the first such reported case in the literature.

## Case presentation

A 56-year-old woman of Han ethnicity was referred to our subspecialty clinic for recurrent SND in the right eye. The patient’s right eye had a history of corneal penetrating injury 20 years ago. After administering anti-inflammatory treatment, the ocular symptoms of pain, light sensitivity, foreign body sensation, and tears vanished. We could not obtain more ophthalmic assessment details of the first visit. After that, the above ocular symptoms occurred in the right eye three to four times a year and were relieved by a 10-day course of medication treatment, such as preservative-free lubrication and anti-inflammation eye drops. In the past year, the ocular symptoms relapsed one to two times a month and the medication could not completely relieve the symptoms. Since detailed medical records at other hospitals were not available, the above clinical history was obtained by inquiry.

**Fig. 1:**
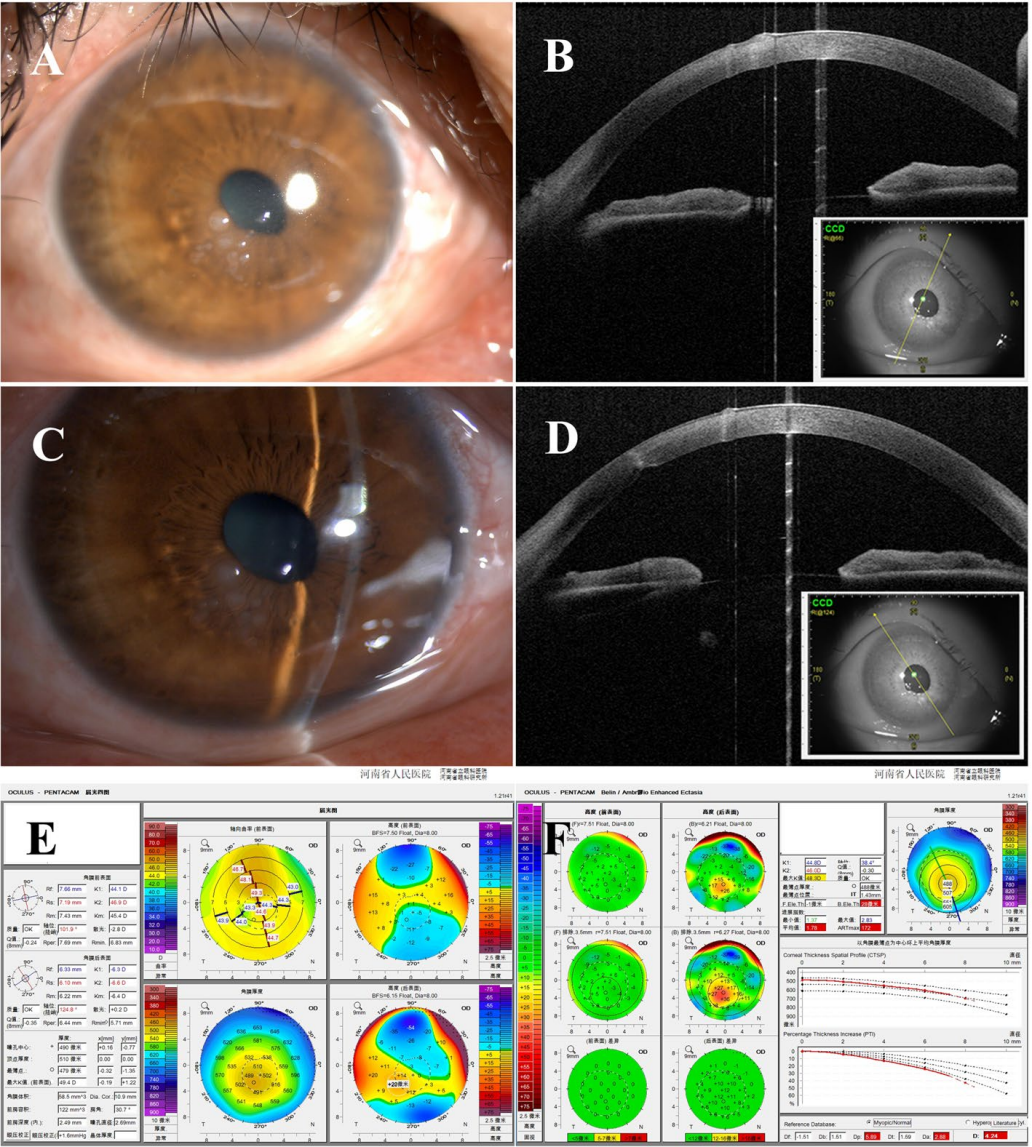
Images Showing the preoperative characteristics of SND accompanying PKC. **A** and **B** Slit-lamp picture and OCT image showing the elevated, whitish-grey subepithelial nodules, corneal scar, and posterior excavation in the mid-peripheral cornea. **C** and **D** Slit-lamp picture and OCT image showing the penetrating corneal scar and posterior excavation. **E** and **F** Pentacam pictures showing the circumscribed elevation of the posterior surface

In December 2019, the patient came to our subspecialty clinic for the new onset of symptoms. Slit-lamp examination revealed elevated, whitish-grey subepithelial nodules in the mid-peripheral cornea, epithelium erosion and edema at the apex of the nodules, remnant corneal scar, and posterior depression (Fig. 1A and C). After a routine examination, the patient was diagnosed with SND accompanied with PKC. The mydriasis test brought out the posterior synechiae at an inferior pupil margin and the overlapping lens opacity. The anterior segment optical coherence tomography (OCT, CASIA SS-1000, Tomey Corporation, Japan) showed prominent bright subepithelial deposits, elevation of the anterior corneal surface (Fig. 1B), localized posterior excavation (Fig. 1D), and penetrating corneal scar (Fig. 1D). The corneal tomography (Fig. 1E and F) demonstrated that the posterior surface overlapping with corneal scar had an abnormal elevation of 28 μm, but the anterior surface was unremarkable with -1 μm. The patient’s other ocular parameters of the right eye are as follows: uncorrected visual acuity: 20/160; best corrected visual acuity (BCVA): 20/63 (− 3.50DS/− 2.50 DC*65); non-contact tonometer: 14 mmHg; axial length: 23.85 mm; and corneal endothelium density: 2509 cells/mm^2^.

Initially, we applied a conservative treatment, including 0.1% sodium hyaluronate eye drops (URSAPHARM Arzneimittel GmbH, Germany), recombinant bovine basic fibroblast growth factor eye drops (Essex Bio-Technology GmbH, China), 0.1% fluorometholone eye drops (Santen Pharmaceutical Co., Ltd. Japan), and bandage contact lens (Bausch & Lomb Incorporated, USA) to manage the latest episode of SND. After a 30-day course, the condition could not be completely relieved. Then we performed a PTK procedure to smooth the corneal surface of the right eye using a ZEISS MEL 80™ Excimer Laser (Carl Zeiss Meditec AG, Germany). Before laser ablation, the delamination of the corneal epithelium was performed using 20% alcohol for about 20 s. After removing the epithelial layer containing degenerative nodules, the resultant underlying surface was slightly irregular. To avoid introducing significant hyperopia drift, 15 μm of PTK was performed. We chose a treatment zone of 7.5 mm that completely covered the lesion area. At the end of surgery, the right eye was covered with a bandage contact lens. Postoperatively, the patient was instructed to use topical antibiotics six times daily until the contact lens was removed. On the third postoperative day, the epithelium healed and the contact lens was removed. The further medical instruction included the topical 0.5% levofloxacin eye drops (Santen Pharmaceutical Co., Ltd. Japan) four times daily, 0.1% fluorometholone eye drops four times daily, recombinant bovine basic fibroblast growth factor eye drops four times daily, and 0.1% sodium hyaluronate eye drops six times daily for 1 month.

**Fig. 2:**
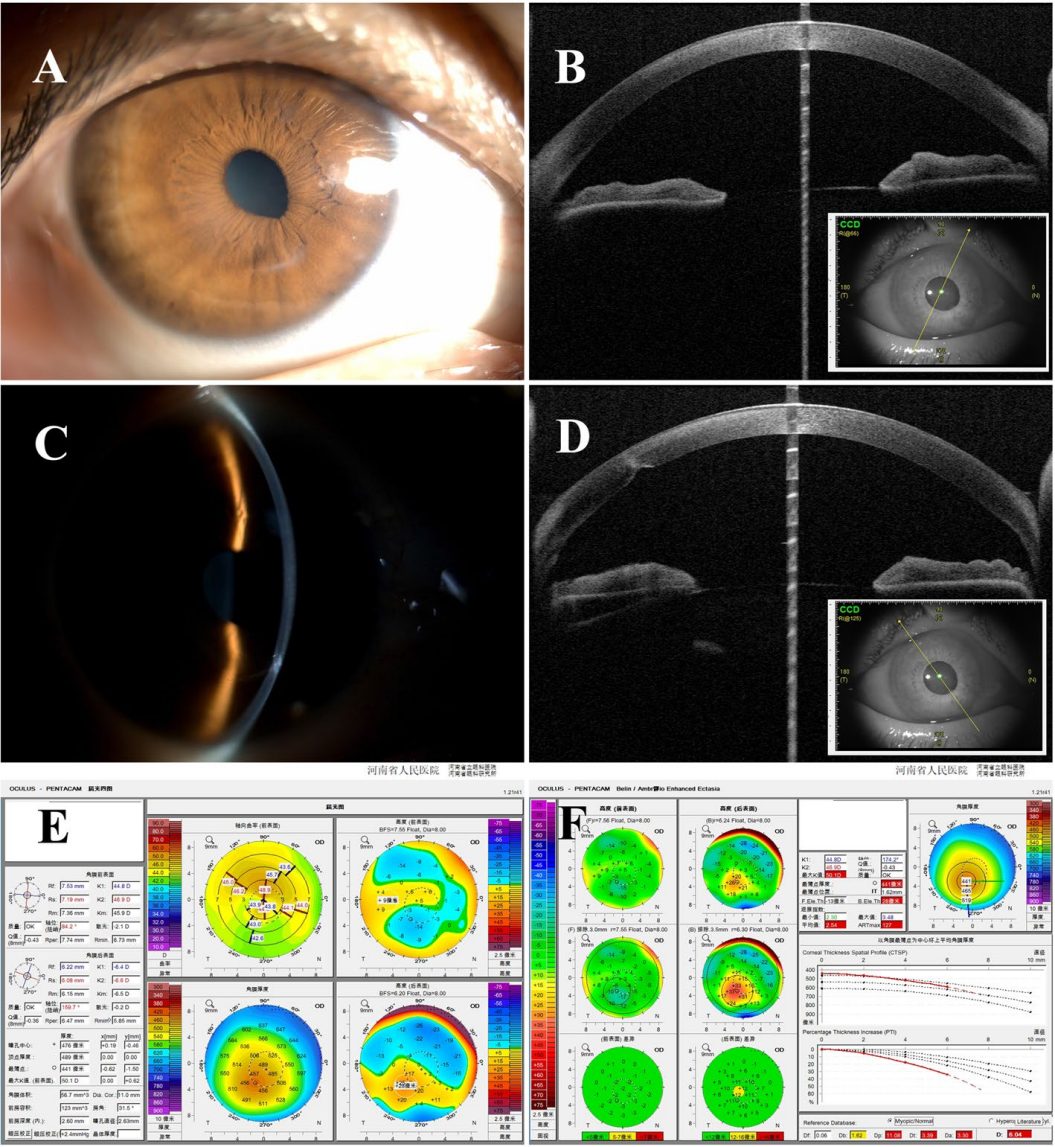
Images Showing the characteristics of SND accompanying PKC at 12 months postoperatively. **A** and **B** Slit-lamp picture and OCT image showing that the elevated nodules disappeared. **C** and **D** Slit-lamp picture and OCT image showing the smooth anterior surface and undisturbed posterior excavation. **E** and **F** Pentacam pictures showing no progressive corneal ectasia compared with Fig. 1F

At the seventh day postoperatively, a slit-lamp examination revealed that the nodules disappeared and the cornea returned to clarity, except for the arc-shaped scar (Fig. 2A and C). The uncorrected visual acuity was 20/63 and BCVA was 20/40 (− 4.50DS/− 1.00 DC*170). From 1 month postoperative to 12 months postoperative, only preservative-free lubrication eye drops were prescribed. At 12 months postoperatively, the cornea remained clear and no SND relapse was recorded (Fig. 2B and D). The Corvis ST (Oculus Optikgeräte GmbH, Germany) examination showed the patient had normal biomechanics with 0.06 of corvis biomechanical index (CBI) and 1.03 of stress-strain index (SSI). The tomography (Fig. 2E and F) showed the elevation of the anterior and posterior surfaces were -13 μm and 28 μm, respectively.

## Discussion and conclusions

The SND and PKC are rare cornea diseases with complicated pathogenesis. To our knowledge, this is the first reported case of SND accompanying with PKC after corneal penetrating injury. Also, a smooth corneal surface was built with no complications and recurrence by the PTK treatment.

The Austrian ophthalmologist, Maximilian Salzmann, first reported the SND in 1925 [8]. Now, the formation of Salzmann nodules remains poorly understood. Multiple risk factors of the SND have been described as trachoma, interstitial keratitis, vernal keratoconjunctivitis, meibomian gland dysfunction, dry eye, pterygium, contact lens wear, ocular trauma, and so on [1]. The location of the nodules depends on the underlying predisposing factors. In our reported case, the nodules were located near the corneal penetrating scar. Previous studies highlighted the inflammation and epithelial damage that multiple risk factors caused by contributing to the formation of SND [1, 9]. The hyaline plaques were detected between the epithelium and the Bowman’s layer in SND cases, and the hyaline degeneration was postulated as a precursor of nodules [3]. Previous histopathological studies also demonstrated the classical histologic features of SND nodules including the epithelium thinning, enzyme imbalance, Bowman’s layer absenting, and abnormal fibrosis tissue [5, 10]. Histopathological evidence implies that an activated immune process and deviant wound repair may lead to an aberrant remodeling of the corneal anterior surface and the formation of nodules. This hypothesis also has the potential to explain the recurring and worsening features of SND.

The PKC is usually congenital and can be associated with other ocular and systemic abnormalities, such as microcornea, synechiae, corectopia, anterior polar cataract, and so on [6]. The trauma has been sporadically proposed as an important cause of acquired PKC, as are an oblique penetrating injury leading to posterior lamellar splitting, destruction of the posterior surface, and keratohematoma [7]. The patient in our report denied any other systematic abnormalities. The slit-lamp and OCT examinations revealed a marked posterior conical protuberance overlapping with the corneal scar. We postulate that the trauma episode may contribute to the inflammatory cascade, localized tissue digestion, and abnormal reconstruction of the posterior surface.

The treatment approaches of SND depend on the size/location of the nodules and severity of the symptoms. The majority of patients respond well to conservative management with topical lubricants and topical anti-inflammatory drugs. The epithelium erosion at the apex of the elevated nodules may associate with the irritation and foreign body sensation. We recommend topical eye drops that promote corneal epithelial healing. Surgical intervention is required in symptomatic patients with vision loss, ocular discomfort, or contact lens intolerance secondary to salzmann nodules. Surgical modalities include superficial keratectomy, PTK, keratoplasty, and some combined procedures such as amniotic membrane transplantation and intraoperative mitomycin-C [1]. The PTK can create a smoother anterior surface than a traditional keratectomy by blade [11]. Previous studies have validated that the PTK is effective and safe for treating SND [12].

In the reported case, the nodules were removed completely without any complications after the PTK procedure. The Corvis ST examination showed that the patient had normal biomechanics. During the one-year follow up, there was no evidence of corneal ectasia.

This case reported, illustrate the rare occurrence of Salzmann’s nodular degeneration accompanied with PKC following a corneal penetrating injury. However, the pathogenesis of post-trauma SND and PKC still requires further exploration. The PTK appears to be a safe and effective option for SND and PKC. The topographic and biomechanical changes of PKC after the PTK procedure require a longer follow-up to verify this.

## Data Availability

The datasets used and/or analyzed during the present study are available from the corresponding author on reasonable request.

## Abbreviations

SND: Salzmann nodular degeneration
PKC: Posterior keratoconus
PTK: Phototherapeutic keratectomy
BCVA: Best-corrected visual acuity
CBI: Corvis biomechanical index
SSI: Stress-strain index
